# Care Decision Making of Frontline Providers of Maternal and Newborn Health Services in the Greater Accra Region of Ghana

**DOI:** 10.1371/journal.pone.0055610

**Published:** 2013-02-13

**Authors:** Ebenezer Oduro-Mensah, Aku Kwamie, Edward Antwi, Sarah Amissah Bamfo, Helen Mary Bainson, Benjamin Marfo, Mary Amoakoh Coleman, Diederick E. Grobbee, Irene Akua Agyepong

**Affiliations:** 1 Ridge Regional Hospital OPD, Adabraka, Accra, Ghana; 2 Greater Accra Regional Health Directorate, Accra Ghana/Wagningen University, Accra, Ghana; 3 Greater Accra Regional Health Directorate, Public Health Division, Accra, Ghana; 4 Greater Accra Regional Health Directorate, Clinical Care Division, Accra, Ghana; 5 Greater Accra Regional Health Directorate, Public Health Division, Accra, Ghana; 6 Ghana Health Service, Accra, Ghana; 7 University of Ghana School of Public Health/University Medical Center Utrecht Str. 6.131, Utrecht, The Netherlands; 8 Julius Center for Health Sciences and Primary Care, University Medical Center Utrecht Str. 6.131, Utrecht, The Netherlands; 9 Greater Accra Regional Health Directorate/University of Ghana School of Public Health, Accra, Ghana; London School of Economics, United Kingdom

## Abstract

**Objectives:**

To explore the “how” and “why” of care decision making by frontline providers of maternal and newborn services in the Greater Accra region of Ghana and determine appropriate interventions needed to support its quality and related maternal and neonatal outcomes.

**Methods:**

A cross sectional and descriptive mixed method study involving a desk review of maternal and newborn care protocols and guidelines availability, focus group discussions and administration of a structured questionnaire and observational checklist to frontline providers of maternal and newborn care.

**Results:**

Tacit knowledge or ‘mind lines’ was an important primary approach to care decision making. When available, protocols and guidelines were used as decision making aids, especially when they were simple handy tools and in situations where providers were not sure what their next step in management had to be. Expert opinion and peer consultation were also used through face to face discussions, phone calls, text messages, and occasional emails depending on the urgency and communication medium access. Health system constraints such as availability of staff, essential medicines, supplies and equipment; management issues (including leadership and interpersonal relations among staff), and barriers to referral were important influences in decision making. Frontline health providers welcomed the idea of interventions to support clinical decision making and made several proposals towards the development of such an intervention. They felt such an intervention ought to be multi-faceted to impact the multiple influences simultaneously. Effective interventions would also need to address immediate challenges as well as more long-term challenges influencing decision-making.

**Conclusion:**

Supporting frontline worker clinical decision making for maternal and newborn services is an important but neglected aspect of improved quality of care towards attainment of MDG 4 & 5. A multi-faceted intervention is probably the best way to make a difference given the multiple inter-related issues.

## Introduction

Between 1990 and 2015, Millennium Development Goal (MDG) 4 calls for a reduction by 2/3 of the under-five mortality rate; and MDG 5 for a reduction by 3/4 of the maternal mortality ratio [1]. Progress towards achievement of MDG 4 & 5 has remained slower than desired in Ghana and other countries of sub-Saharan Africa [2], [3], [4], [5], [6], [7], [8]. Maternal and neonatal deaths are caused by a complex interaction of economic, financial, social, cultural and service access and quality factors. In Ghana, there is reasonable access to Antenatal Care (ANC) with about 95% of women 15 – 49 years receiving ANC from a skilled provider (value is 95.7% [7] in the Greater Accra region). Delivery by a skilled provider is lower with a national average of 59% [9], and 84.3% in the Greater Accra region [7]. A free maternal care policy was introduced in four regions of Ghana in 2003, and subsequently in 2005 to the whole country to reduce financial access barriers. Assessment suggested that though the policy had led to increases in institutional deliveries, institutional maternal mortality rates had not decreased [10], [11], [12], [13]. Quality of service within facilities remained problematic, and is considered to be partly responsible for the persisting high national average maternal mortality rate (MMR) estimated at 451 per 100 000 live births and neonatal mortality rate (NMR) of 30 per 1,000 live births [14], [15], [16]. Gaps identified in the quality of care given to pregnant women when they use health facilities include decisions on management, as well as information given to women by frontline providers [17].

As providers interact with clients, they continually make decisions about client needs and the appropriate service to provide. Potentially important supports for this process are availability and use of evidence based / informed decision making guidelines and tools that improve and make performance more consistent, by reducing guesswork and promoting compliance with standards [18]. In evaluating the effectiveness of alternative training models and other performance improvement factors on the quality of maternal care and client outcomes through the safe motherhood program in Ghana, it was found that about 21% of intervention and comparison facilities still did not have clinical management protocols and guidelines at the front line [19]. However beyond availability is whether when available, evidence informed guidelines are extensively used and important drivers of frontline provider care decision making. A cross country comparison of maternal health guidelines in Burkina Faso, Ghana and Tanzania concluded that format of guidelines and implementation strategies rather than poor quality of content or lack of evidence was the major barrier to positive impact of guidelines on quality improvement [20]. Clearly there are other important factors to clinical decision making than just evidence based guidelines [21]. An ethnographic study in the UK found that primary care clinicians (general practitioners and practice nurses) derived their individual and collective health care decisions from “mind lines” or “collective reinforced, internalized tacit guidelines” rather than practice guidelines [22]. The importance of tacit knowledge in decision making is documented from other studies [23].

The current study focused on understanding the “how” and the “why” of care decision making for clients needing maternal and newborn health services by frontline providers (doctors, midwives, public and community health and family planning nurses) in the Greater Accra region, one of the ten administrative regions of Ghana. Its estimated population of almost four million is 15% of Ghana’s population. It is almost 90% urban, well above the national average of about 45%. It has one of the highest population growth rates in the country despite having the lowest total fertility rate in the country (2.5%), well below the national average of 4% [24]. Migration in from poorer regions of the country is a major contributor to the high population growth. This massive rural urban drift may account for the fact that it is the only region in Ghana that showed a rise in poverty levels in the 2005/06 Ghana living standards survey [25]. The percentage of the population living below the poverty line that had dropped in the region from 23% in 1991/92 to 4% in 1998/99; rose to 11% in 2005/06. Even with this rise it remains the region with the lowest poverty levels in Ghana, well below the national average of 52%. The region had a pregnancy related mortality ratio of 448 (95% CI 268 – 578) per 100,000 live births in the 2007 Ghana Maternal Health survey compared to the national average of 416 (95% CI 313 – 520) [26]. Neonatal mortality in the region for the 10 year period before the 2008 Ghana Demographic and Health survey was 21 deaths per 1,000 live births [27], as compared to the national average of 27/1000 live births.

## Objectives

The objective was to explore “how” and “why” care decision making for maternal and newborn care by frontline providers (doctors, clinical, public and community health nurses and midwives) is done; and interventions that would be most appropriate to support and improve the quality of care decision making.

**Figure 1:**
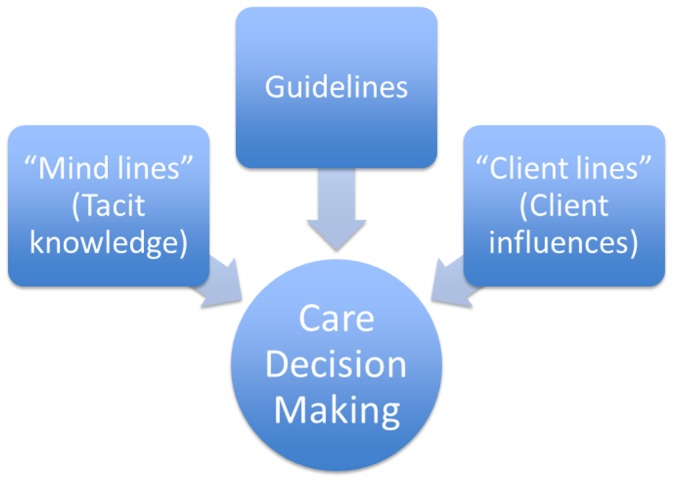
Initial Conceptual framework. “How” and “Why” care decisions are made by frontline providers of maternal and newborn services

Based on a non-exhaustive review of the literature [28], [29], [30], [31], [32], [33] as well as a discussion of the observations and experiences of the members of the research team who had worked with frontline providers of reproductive and neonatal health services in the study setting, the study started off with a simple conceptual framework that theorized that care decision making of frontline providers was influenced by clinical guidelines, “mind lines” or tacit knowledge and “client lines” or client influences related to the preferences and pressures of the client and the wider family and community, including social, religious and cultural values and beliefs. This is shown as figure 1.

## Methods

A mixed method, exploratory, cross sectional and descriptive case study of frontline provider decision making was carried out between July and December 2011 in the Greater Accra region of Ghana. Data collection involved a desk review of maternal and child health evidence based guidelines and decision making tools available in Ghana, focus group discussions and administration of a structured questionnaire with closed and open ended as well as observational checklist type items for actual guideline availability to frontline staff. The Ghana Health Service Ethics Review Committee gave approval for the study [34]. Written informed consent was obtained from all participants. No potential respondent opted out of the study during the study period.

For the desk review, a search was conducted of records and libraries of Ghana Health Service and development partner agencies supporting maternal and neonatal health. Interactions with Agency representatives to explain what was being searched for was used to selectively target the search. “Guidelines” were defined as written materials whether books, booklets, charts, leaflets, posters or simple aids like diagnostic wheels; providing information, directions and guidance to aid frontline staff in clinical decision making and case management of clients presenting for maternal and newborn health care services whether preventive, promotive or curative.

The Greater Accra region contains the capital city of Ghana, Accra. Of the 10 local government districts in the region at the time of the study, two were metropolitan and six municipal and contained almost 80 – 90% of the population. The remaining two were rural. For the primary data collection, a district was randomly selected from each of the 3 categories of metropolitan, municipal and rural by simple ballot. This categorization was used because observation suggests that urbanization affects health service availability. Staffs are more reluctant to be posted to the rural districts and social and communication infrastructure such as road networks and internet access are more limited. Though there are no constraints with staff accepting postings to the municipal districts, population growth in these areas has outstripped infrastructure. The municipal populations are therefore generally less well served with health facilities than the metropolitan. In the metropolitan district, the size of the population (almost two million) is such that service delivery is further decentralized to the sub-metropolis. Therefore one sub-metropolis was selected randomly by simple ballot from amongst the six sub-metropolitan districts. District managers, midwives, community health officers, public and community health nurses, medical assistants, and doctors were invited to participate in Focus Group Discussions (FGD). Participants were conveniently selected based on their availability and willingness to participate. The FGD were structured so as to not disrupt service delivery or provision of care to clients. Staff run eight to twelve hour shifts depending on category; and also have days off. Staffs were invited to participate in the FGD during their off period. Reimbursement of transport to and from the FGD venue was provided.

The Focus group discussions (FGDs) were conducted to gain perspectives from these staff on how and why they make their client care decisions, needs and potential components of an intervention to support care decision-making. Five FGD sessions, each lasting an average of two hours with between 6 to 10 participants per session were held with staff grouped into those providing similar kinds of service as follows: Heads and Managers of facilities providing RCH services; Midwives; Family Planning, Public and Community Health Nurses; Medical Doctors and Medical (Physician) Assistants.

Notes were taken during the FGD, in addition to recording and transcribing the interviews. All FGD data analysis was done manually looking for themes, commonalities and contrasts using an inductive approach. Exploration was done for the themes in the starting framework (figure 1) as well as themes that had not been captured in the starting framework, but were emerging in the interviews. The interviews were stopped when the analysis was not yielding any new insights beyond what previous FGD had already yielded. Analysis of each FGD was done by at least two team members who then discussed and compared notes. The analysis was discussed with all team members before concluding.

The results of the FGD informed the revision of the draft of a structured questionnaire with closed and open ended as well as observation checklist items for use with frontline staff to obtain more quantitative data as part of triangulation. The questionnaire was pretested with frontline staff in a non-participating sub-metropolis. The final questionnaire was interviewer administered. Variables on which data was collected included availability of centrally developed care decision making support tools and guidelines, locally adapted or developed tools and guidelines, actual use, familiarity with guidelines content, management of referrals, common communication mechanisms and channels and their ease and convenience of use, including use of mobile phones and internet and access to expert advice and information.

Facilities in the study areas where interviews were administered to frontline staff were three district level hospitals, two polyclinics, one health center and two urban and six rural Community Health Planning and Services (CHPS) zones. All those at post during the week of the interviews were interviewed. If staff were busy e.g. with an emergency, interviewers returned at a more opportune time. Fifty (50) staff members providing antenatal, delivery, postnatal and newborn care services and fifteen (15) staff members providing family planning services were interviewed. The interviews were conducted at their points of service delivery, except for the Community Health Nurses working in CHPS zones in the community who had to be tracked down within the community. Data was entered in Excel and then imported to and analysed in STATA®. The interviews provided quantitative data, which is presented simply as numbers of responses and percentages.

## Findings/Results

### The “How” and “Why” of Frontline Provider Decision Making - FGD Findings

**Figure 2:**
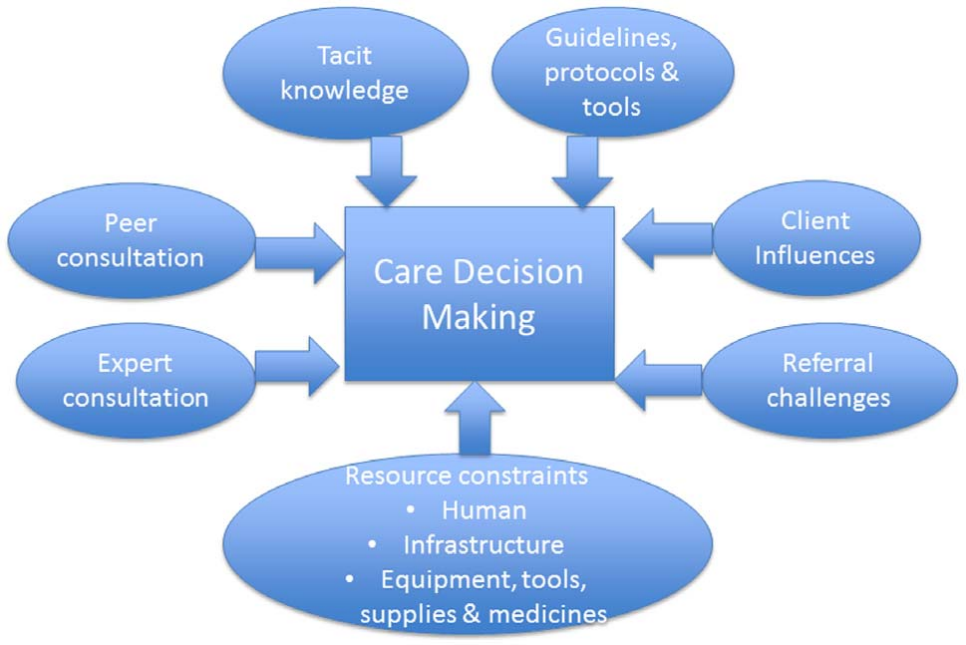
Final Conceptual framework based on data analysis. “How” and “Why” care decisions are made by frontline providers of maternal and newborn services.

In the FGD, frontline providers raised a series of factors, explaining the “how” and the “why” of their decision making. The information from the FGD led to an expansion of the simple starting theoretical framework as, summarized in Figure 2 and explained below.

All the three factors we theorized in our starting framework as important in decision making emerged as such during the FGD. **Tacit knowledge** or “mind lines” [28] was a common “how” of care decision making. Frontline providers indicated that tacit knowledge is acquired over time from both pre-service and in-service training, and the “*rich experience”* and skills acquired on the job.

*“You don’t want to have a maternal death – that is the first thing that flashes through the mind; that this patient must not die, and that gives you the drive.…after you’ve thought of that, then you look at the patient’s clinical condition. Then, all the stuff that you have in your head begins to pour….”* (Physician in charge of a district hospital)

Also influencing how decisions were made was the availability and content of **evidence based guidelines, protocols and tools**. Respondents said they referred to these, especially when not sure what their next step in management had to be. They preferred protocols tailored to the level of service provided at the facility. Handy sized and user friendly rapid assessment tools such as wall charts on management protocols were mentioned as more used on a routine day to day basis than bulky comprehensive documents, which were used as references.

“**Client lines**” as in our starting theoretical framework came out in the FGD as influencing clinical decision making. In choice of a family planning method, staff indicated that they sought to understand these influences and accommodate them – to the extent possible – in decision making. In other cases, especially where providers felt the client’s life was at risk, client refusal to accept some decisions could create provider frustration and life threatening dilemmas.

*“….some of the religious and societal interference on decisions taken for patients…. For example, a pastor tells a patient that he has seen a vision that after the operation she would die. It is a big challenge because even for (the sake of) their babies they wouldn’t allow you to do anything for them….….”* Midwife

In addition to the three factors in our framework, other major factors affecting decision making emerged in the FGD necessitating a revision of the framework. The ability to obtain **expert opinion** influenced care decision-making. Communicating with respected senior colleagues and authorities or sometimes peers to ask opinions was done in the form of phone calls and text message, and occasionally by email if face to face was not possible. For on-the-spot decisions, phone calls and text were preferred as faster, as internet access is unavailable at most work stations. On a more long-term basis, face-to-face meetings and discussions supported **peer learning and consultation** and enhanced tacit knowledge.

Challenges surrounding **the referral process** emerged as a consistent theme influencing decision making and sometimes making the decision to refer a very difficult one. They included inadequacies of ambulance services such as insufficient numbers of vehicles, and delays due to traffic; waiting time to see a doctor, lack of beds, or on-duty doctor, resulting in the need for clients to be transferred elsewhere; client financial constraints, whether for payment of travel costs, or supplies not covered by the National Health Insurance Scheme; and the quality of the client reception at the referral point, including relationships between facility staff, as well as towards referred clients.

Several instances were cited where clients refused **referrals** for logistic and service responsiveness reasons such as the travel distance, time and money involved as well as the uncertainty of the kind of reception they would get at the receiving hospital.

*“…….patients refuse to go on referral. …. They don’t understand why you are there and you ask them to go to another hospital……. some will not go at all. Some will go but will keep long (delay) before they go….Some …. will tell you ‘Okay, give us money so that we go’.”* Nurse midwife

Also influencing care decision making were **health system resource constraints** such as availability of staff, medicines, supplies such as oxygen and equipment, **management issues** (including leadership and interpersonal relations among staff). A repeated concern was the inadequate numbers of senior professionals who frontline workers could confer with whether by phone or face to face when confronted with a decision making challenge. Some frontline workers thought they could very easily have managed some cases they have had to refer if only someone had been accessible to talk and guide them through the appropriate management.

The remote location of some facilities was also cited as a challenge in decision-making by staff from the rural districts. It affected what was the feasible decision as opposed to the optimal decision in a given situation. An emergency case whose chances of survival depended on needed care in under an hour might have to be managed as best as possible in the local facility despite limited capability; if the shortest time to get to a higher facility was several hours at best.

Importantly, these factors often influenced clinical decision making interactively rather than independently. Thus for example, information from discussion with a peer could go to enhance the tacit knowledge pool; and referral challenges could be influenced by the availability or otherwise of expert consultation. This is conceptualized in the theoretical framework by the lines linking the boxes into a closed whole.

#### Evidence Based guidelines and decision making tools – Desk review

The desk search for frontline provider evidence based guidelines and decision making support tools related to maternal and newborn care endorsed for use in Ghana by policy makers revealed those listed below, which are further explained in box S1 attached:

1. Ghana Health Service (2008) National Safe Motherhood (SM) service protocol.
2. Ghana Health Service (2007) The National Family Planning Protocols manual.
3. Reproductive Health Service Policy and Standards (2003).
4. Ministry of Health – Ghana National Drugs Program (2011) Standard Treatment Guidelines (STG).
5. Local adaptation of the WHO Integrated Management of Neonatal and Childhood Illness (IMNCI) manual and chart booklets by Ghana Health Service.
6. Ghana Health Service (2006) Prevention and Management of Unsafe Abortion: Comprehensive Abortion Care Services, Standards and Protocol, June 2006.
7. Improving access to quality care in Family Planning (FP), Medical Eligibility Criteria for contraceptive use (MEC Wheel) (2008).

### Findings from Frontline Staff Interviews

Data from the interviews is summarized as tables 1, 2, 3, 4, 5. A single asterix (*)means sum either greater or less than 100% due to elimination of decimal points. A double asterix (**) means the family planning clinics in the study did not have any locally modified or developed guidelines.

**Table 1:** Demographic Data of Respondents to questionnaire survey on Reproductive and Neonatal Health (RNH) service provision.

Variables	Rural district			Municipality			Sub-metropolis			Total			% of all respondents
	MCH staff	FP staff	Total	MCH staff	FP staff	Total	MCH staff	FP staff	Total	MCH staff	FP staff	Total	
	N = 16	N = 6	N = 22	N = 16	N = 5	N = 21	N = 18	N = 4	n = 22	N = 50	N = 15	N = 65	
Mean age in years (SD)	35.8 (10.4)	39 (12.6)		44.4 (12.0)	50 (5.7)		45.8 (11.0)	50.5 (6.4)		42.7 (11.7)	45 (10.7)		
Male	1	0	1	1	0	1	1	0	1	3	0	3	5%
Female	15	6	21	15	5	20	17	4	21	47	15	62	95%
**Status of facility** *													
District hospital	8	2	10	3	0	3	8	2	10	19	4	23	35%
Polyclinic/Health centre	4	3	7	12	3	15	8	2	10	24	8	32	49%
CHPS zone /compound	4	1	5	1	0	1	2	0	2	7	1	8	12%
Private	0	0	0	0	2	2	0	0	0	0	2	2	3%
**Professional Grouping** *													
Medical Doctor	1	0	1	1	0	1	1	0	1	3	0	3	5%
Physician Assistant	1	0	1	1	0	1	1	0	1	3	0	3	5%
Nurse	14	6	20	14	5	19	16	4	20	44	15	59	91%

**Table 2:** Responses to use of and Types of Aids to Decision Making among Frontline Service Providers of Reproductive and Neonatal Health Services.

Variables	Rural district			Municipality			Sub-metropolis			Total			% of all respondents
	MCH staff	FP staff	Total	MCH staff	FP staff	Total	MCH staff	FP staff	Total	MCH staff	FP staff	Total	
	N = 16	N = 6	N = 22	N = 16	N = 5	N = 21	N = 18	N = 4	n = 22	N = 50	N = 15	N = 65	
**Do you regularly use any tools as aids in your daily clinical decision-making?**													
Yes	16	6	22	16	5	21	16	4	20	48	15	63	97%
No	0	0	0	0	0	0	2	0	2	2	0	2	3%
Which of these do you use to aid your daily clinical decision-making (indicate “Yes”):*													
Printed Protocol /Guidelines	16	6	22	15	5	20	15	4	19	46	15	61	94%
Charts	16	6	22	14	5	19	13	4	17	43	15	58	89%
Workshops	16	5	21	15	5	20	13	4	17	44	14	58	89%
Expert advice	16	6	22	14	5	19	13	4	17	43	15	58	89%
Telephone	14	3	17	14	3	17	13	2	15	41	8	49	75%
How often do you use locally modified / developed guidelines**													
Never or rarely	1			0			1			2			4%
Occasionally, frequently or always	12			14			17			43			86%
Non-respondents	3			2			0			5			10%

**Table 3:** Responses to the use of Information Communication Technology as Aids to Clinical Decision Making.

Variables	Rural district			Municipality			Sub-metropolis			Total			% of all respondents
	MCH staff	FP staff	Total	MCH staff	FP staff	Total	MCH staff	FP staff	Total	MCH staff	FP staff	Total	
	N = 16	N = 6	N = 22	N = 16	N = 5	N = 21	N = 18	N = 4	n = 22	N = 50	N = 15	N = 65	
**Do you have access to an official phone at the point of service delivery when you need to call for help in an emergency?**													
Yes	9	2	11	14	2	16	16	3	19	39	7	46	71%
No	7	4	11	2	3	5	2	1	3	11	8	19	29%
**Are you always able to use your mobile phone to call for help when faced with an emergency?**													
Yes	16	6	22	16	2	18	15	4	19	47	12	59	91%
No	0	0	0	0	3	3	3	0	3	3	3	6	9%
**Do you use text messaging?**													
Yes	12	3	15	13	4	17	13	1	14	38	8	46	71%
No	4	2	6	3	0	3	5	3	8	12	5	17	26%
Non-respondents	0	1	1	0	1	1	0	0	0	0	2	2	3%
**If yes, how frequently do you send/receive text messages?**													
At least once a day	1	0	1	3	0	3	3	0	3	7	0	7	11%
Several times a week	8	2	10	8	2	10	4	0	4	20	4	24	37%
Occasionally	3	1	4	2	2	4	6	1	7	11	4	15	23%
**Have you ever sought advice from a senior colleague/expert outside of your facility when faced with making a critical decision in an emergency?**													
Yes	12	5	17	16	5	21	10	4	14	38	14	52	80%
No	4	1	5	0	0	0	8	0	8	12	1	13	20%
**Do you have ready access by phone to any obstetrician either within or outside your facility?**													
Yes	12	3	15	16	3	19	13	0	13	41	6	47	72%
No	4	3	7	0	2	2	5	4	9	9	9	18	28%
Do you use the internet?*													
Yes	7	2	9	8	2	10	8	0	8	23	4	27	42%
No	9	3	12	8	3	11	10	4	14	27	10	37	57%
Non-respondents	0	1	1	0	0	0	0	0	0	0	1	1	2%

**Table 4:** Responses to the use of face-to-face meetings as aids to clinical decision making among frontline providers of reproductive and neonatal health services.

Variables	Rural district			Municipality			Sub-metropolis			Total			% of all respondents
	MCH staff	FP staff	Total	MCH staff	FP staff	Total	MCH staff	FP staff	Total	MCH staff	FP staff	Total	
	N = 16	N = 6	N = 22	N = 16	N = 5	N = 21	N = 18	N = 4	n = 22	N = 50	N = 15	N = 65	
**Have you attended any workshop related to reproductive and child health this year?**													
Yes	5	3	8	9	5	14	10	0	10	24	8	32	49%
No	11	2	13	7	0	7	8	4	12	26	6	32	49%
Non-respondent	0	1	1	0	0	0	0	0	0	0	1	1	2%
**Do you have regular clinical meetings at your department/unit?**													
Yes	14	5	19	15	5	20	13	4	17	42	14	56	86%
No	2	0	2	1	0	1	5	0	5	8	0	8	12%
Non-respondents	0	1	1	0	0	0	0	0	0	0	1	1	2%
If yes, how often:*													
Once a week	4	4	8	5	0	5	2	0	2	11	4	15	23%
Twice a month	2	0	2	5	1	6	3	2	5	10	3	13	20%
Once a month	7	1	8	4	4	8	7	2	9	18	7	25	38%
Less than once a month	0	0	0	1	0	1	0	0	0	1	0	1	2%
Thrice in a year	0	0	0	0	0	0	0	0	0	1	0	1	2%
Occasionally	1	0	1	0	0	0	1	0	1	1	0	1	2%
Non-respondents	2	1	3	1	0	1	5	0	5	8	1	9	14%

**Table 5:** Responses to availability of guidelines at the point of service delivery.

Variables	Rural district			Municipality			Sub-metropolis			Total			% of all respondents
	MCH staff	FP staff	Total	MCH staff	FP staff	Total	MCH staff	FP staff	Total	MCH staff	FP staff	Total	
	N = 16	N = 6	N = 22	N = 16	N = 5	N = 21	N = 18	N = 4	n = 22	N = 50	N = 15	N = 65	
**Do you have any of these guidelines related to MCH with you here today kept at the point of service delivery (question asked only of the 50 MCH staff)**													
Yes, Safe Motherhood protocol	9			12			12			33			66%
Yes, Standard treatment guidelines	15			14			10			39			78%
Yes, others	10			2			0			12			24%
**Do you have any of these guidelines related to FP with you here today kept at the point of service delivery (question asked only of the 15 FP staff)**													
Yes, Family planning protocol		6			4			4			14		93%
Yes, WHO Guidelines on FP		4			2			3			9		60%
Yes, eligibility criteria wheel		4			2			3			9		60%
Yes, flipcharts on FP		6			5			4			15		100%
Yes, flip charts on abortion		1			0			2			3		20%

The majority of the staff interviewed providing general maternal and newborn care services were nurses (44/50). Of the remaining 6, three were doctors and 3 were physician assistants. All the 15 family planning service delivery staffs were public and community health nurses.

Almost all the providers of general maternal and newborn care services (96%) said they regularly used some aids in their daily clinical decision making. Printed protocols and guidelines were the most commonly selected (96%). However workshop materials (92%), expert advice (90%) and telephone calls for advice (85%) were also frequently selected as aids in daily decision making.

Observation of guidelines actually available at the service delivery point showed that the commonest were the safe motherhood protocol (66%), and the standard treatment guidelines (78%). Twelve of the staff (24%) had other reproductive and child health protocols. Only one respondent had the IMNCI protocol. The year of publication for the safe motherhood protocol ranged from 1999 to 2008; and that of the standard treatment guidelines from 2002 to 2011.

Fourteen (14) out of the 15 family planning nurses had the family planning protocol, 9 had the WHO guidelines on Family Planning whiles 11 had the Medical Eligibility Criteria Wheel.

The majority of respondents (80%) said they had access to various local (institutionally) modified or developed guidelines and protocols e.g. management of postpartum haemorrhage, management of pre-eclampsia / eclampsia, management of antepartum haemorrhage, active management of the third stage of labour, neonatal resuscitation, managing prolonged labour etc. These guidelines were all modifications of the existing national or international protocols, and were modified to either suit the level of care provision or convert them into ‘easier to refer to’ charts. Compared to the safe motherhood protocol, 88% of respondents found the modified guidelines easier to use.

Only 39 out of the 50 providers of general maternal and newborn care services indicated they had access to a facility or official phone at point of service delivery to use for work related calls. Personal mobile phone ownership on the other hand was universal. Forty seven out of the 50 staff indicated that they were always able to use their mobile phones to call for help when faced with an emergency.

Seventy eight percent of staff (38/50) used text messaging on their mobile phones for any reason – whether work or non-work related. Of the staff who used text messaging (N = 38); the majority (20) used it several times a week, 11 occasionally and 7 at least once a day. Internet use was lower, with only 23/50 (46%) of staff indicating they used the internet. There was no noticeable difference between the rural, municipal and urban staff.

Consulting senior colleagues for advice when faced with critical decisions was common (76%). There were no specialists in the peripheral primary care units, which were the focus of this study. However, 82% of staff indicated that they were able to access an obstetrician for advice by phone if they needed to.

Just about half of respondents (48%) had attended an in-service training on reproductive and child health in the year 2011, but eighty four percent (84%) had regular clinical meetings at their facility, though the frequency of these meetings varied from facility to facility.

The average travel distance between referring and receiving facilities was 52 minutes, with the longest being two hours. Reasons for preferentially choosing one referral facility over the other varied, and included previous experiences with reception at the receiving facility, as well as the perception of how equipped or ready the receiving facility was perceived to be in managing emergencies. Regarding challenges with referrals, 72% of respondents cited lack of transportation as a major barrier, as against 62% and 54% for patient’s refusal to be referred and poor reception at the receiving facility respectively.

### Supporting Frontline Worker Care Decision-making

All respondents in the FGD were enthusiastic about the idea of putting in place some kind of intervention to support care decision-making. Importantly, frontline workers felt that any intervention ought to address the multiple challenges at the same time. For emergency cases, the availability of working phones to make **phone calls** was considered important as a way of reaching experts. A help centre/hotline model where staff could call in and be linked to an expert to discuss difficulties and get feedback was suggested. **Text messaging** was suggested as a way of providing daily tips, which could be of use to front-line staff. **District linkages** were also mentioned as a way of supporting decision-making in a systemic way. Specialists could be assigned to a catchment area, and the linkages would cascade, such that community and sub-district level staff could call up to the district and districts could call across or up to the regional level as well. The importance of a feedback loop to the callers was also raised.

Use of the **internet** was considered to be useful on a long-term basis in terms of information provision to enhance knowledge. Protocol websites, medical journals, and social networking were all mentioned as ways of using the internet. Additionally, the networking of facilities to one another was deemed a useful way to ensure continuity of patient information for referral.

**Face-to-face meetings**, ‘in-house’ workshops, mortality conferences and team discussions were also mentioned as ways of sharing information and learning among staff. Respondents noted the need for a regular frequency of such meetings.

Other considerations raised included the fact that any intervention should be cost-effective to the end-users, and should also have a capacity strengthening component. Regarding capacity strengthening it was felt that there needed to be better methods of ensuring team-wide opportunities of information sharing as not all staff had opportunities to attend in-service trainings. Furthermore, respondents felt that an emphasis should be placed on building confidence and competence, and that training should be practical and not only theoretical. Continuous self-education was mentioned as a desired objective.

Also mentioned were making protocols and guidelines readily available at the workplace, providing periodic refresher training, providing essential logistics to work with, access to expert opinion, availability of an efficient ambulance service, easy accessibility of referral centres, increase in number of essential staff / personnel at the point of service delivery, accessibility of specialists by phone and provision of official phones at points of service delivery.

At the end of the structured interviews, each staff interviewed was asked a concluding open ended question: “List three ways in which you believe you can best be assisted to make the right decisions at the point of service delivery when faced with an emergency and uncertain as to what to do”. The suggestions raised are closely related to the suggestions above from the FGD as the analysis of their categorized responses in table 6 below shows. Since staff could make up to three responses, the total number of suggestions (171) is greater than the sample size of 65. The most frequent response was for printed guidelines and protocols, closely followed by access to senior and more experienced and skilled colleagues to provide advice. Next was adequate provision of equipment tools and supplies. These were generally very basic items taken for granted in more developed economies such as oxygen cylinder, speculum, lithotomy couch, ultrasound scan, computer, internet access etc. In one case the lack of a theatre for emergency obstetric procedures was mentioned. The fourth most frequent set of responses were for work telephones. Initially work telephones were classified with equipment, tools and supplies, but observing the frequency of the requests for that particular item, it was separated out.

**Table 6:** Summary of responses to the open ended question: “List three ways in which you believe you can best be assisted to make the right decisions at the point of service delivery when faced with an emergency and uncertain as to what to do”.

Category of response	No. ofresponses	% of responses (N = 171)	% of staff (N = 65)	Examples of responses in this category
Protocols and Guidelines	37	22%	57%	13 out of the 37 responses in the category specifically asked for “printed protocols”; 12 wanted “charts”; most of the rest mentioned specific protocols they wanted; a few just wanted unspecified “guidelines' or “protocols”.
Consultation - Senior colleague/Expert	36	21%	55%	“Access to the supervisor and calling them”; “advice from senior colleagues”; “an experienced midwife can be contacted”; “easy access to phone for calling a senior colleague for help”; “expert advice”
Equipment tools andsupplies	26	15%	40%	“Adequate provision of logistics”; “all equipment should be available”; “computers with internet access”; “basic emergency items should be made available e.g. oxygen cylinder”
Provide a Telephone/telephone	15	9%	23%	“Provision of an office phone”; “provision of official phones”; “telephone”
Periodic refresher training	13	8%	20%	“attend regular workshop to upgrade knowledge”; “regular in-service training”; “training programs”; “workshops”
Referral transportation/Ambulance	14	8%	22%	“ambulance availability”; “cash to assist clients transportation”; “means of transport for referral”
Other referral related	6	4%	9%	“prompt communication with referral centres”; “quick referral”
Staffing	11	6%	17%	“specialists care”; “specialists availability”; “a need for a doctor”; “more staff”; “doctors should be available and easily approachable”; “there should be an obstetrician on call 24/7”
Peer consultation	7	4%	11%	“advice from colleagues”; “by calling a colleague”
Client participation	2	1%	3%	An inauguration ceremony should be done to make community members aware of the Community Based Health Planning and Services (CHPS) compounds; The Community Health Committee should be inaugurated
Other /unclear howto categorize	4	2%	6%	“call for help”; “career progression”; “effective communication”; “regular facilitative supervision”

The individual staff responses reflect the responses from the FGD, thus strengthening the validity of the conclusions of the FGD analysis. Additionally, they enable some quantification of the relative importance of the different expressed areas of need for supporting and strengthening decision making by frontline providers of MDG 4 and 5 related services in the study area. The responses also show the inter-relatedness of the issues. Thus the requests to be provided with telephones for work related calls were generally related to the request to facilitate the ability to quickly consult an expert for advice when in a dilemma. Though almost all staff had personal phones, using those phones for work related calls meant piling up phone bills to be paid for personally out of pocket for official work calls. The request for “work telephones” was effectively a request for the institution to bear the cost of work related calls.

## Discussion and Conclusions

Understanding frontline worker care decision making for maternal and newborn client services is important in developing interventions to support attainment of MDG 4 and 5. Given that delays within the facility are closely related to decision making and one of the contributory factors to maternal mortality [35], it is important to pay more attention to this area. Our findings suggest several potential innovative interventions that could reduce this third delay by supporting and improving care decision making within the health facility.

Two general points arise from our study, concerning any interventions in this area. Firstly, there are multiple issues affecting the decision making process and they interact rather than work in isolation. Any intervention should therefore be multi-faceted. Secondly, it should also take into account how to work with and around major health system constraints such as staffing inadequacies and referral system challenges.

Several specific ideas for intervening arise out of our observations. Firstly, ensuring near universal availability of up to date evidence based guidelines and protocols is important. The importance of guidelines and protocols is already recognized and in Ghana, where this study was conducted, the Ministry of Health and Development partners already spend money each year in printing voluminous protocols and guidelines which eventually find their way into desks and shelves. However, the effective availability of these protocols and guidelines is still inadequate and not all staff in this sample had access to them. Furthermore, there is the need to take a critical look at which form of guidelines will be truly useful to frontline health workers. Our findings suggest that there must be a balance between detailed voluminous reference information as against simple charts and other similar rapid consultation decision making aids which clearly define management steps in emergencies. Frontline worker responses suggest that whilst appreciating the voluminous guidelines in circulation for reference purposes, more of the quick reference simple charts need to be designed and produced in quantities such that each staff has them readily available.

It is also important to address the infrastructure, tools and supplies constraints of frontline health workers, many of which are very basic. The ability to perform is not only a function of knowledge and information (tools and guidelines) but also of an enabling environment. The referral challenge also stands out. Transportation related delays in the community to reach a skilled attendant are recognized. Our study suggests that even after reaching a skilled attendant, transportation related delays for mothers who need a higher level of care are also an important issue.

We also see a need to more critically explore the potentials of modern information technology, specifically phones and the internet. The near universal ownership and use of mobile phones in the study setting suggests that frontline provider decision making support interventions that employ mobile phones can be potentially useful. An evaluation of an intervention that used mobile phone text messages as reminders found that it improved the adherence of Kenyan health workers to treatment guidelines [36]. There are however very few such studies available, and a recent review of the potential to improve maternal health services through the use of mobile phones [37] concluded that robust studies in this area are lacking – making this a clear gap area for future work.

In devising interventions that use telephones, it must be kept in mind that there are costs to communicating by mobile phone. Staff in this study despite their high personal mobile phone ownership expressed a need for “official phones”. The request for “official phones” is so that the cost of communication does not end up being borne by the staff – out of often already too small salaries. Alternatively, where members of staff are being asked to use their personal mobile phones for work related communication; some means must be devised to reimburse them for the cost. Though internet use was lower, it was still being used and it is definitely worth exploring how to harness and effectively use it. This is especially so given that in some cases internet access was part of the list of “equipment, tools and supplies” frontline workers wanted. The advent of smart phones makes a future link between mobile phone ownership and internet access and use in supporting decision making a definite possibility.

The importance of telephones and the internet in clinical decision making is closely related to the need to facilitate communication between frontline providers and peers and experts who can be quickly reached to provide inputs into decision making. Setting up interventions that give frontline staff better access to expert advice is critical, especially in the human resource constrained environments in which the staffs in this study work.

In conclusion, we make a plea that more attention and resources needs to be provided to research, implementation, monitoring and evaluation of effective interventions to support frontline provider decision making and management of mothers and newborns if attainment of MDG 4 and 5 goals are to be accelerated in sub-Saharan Africa.

### Limitations of the Study

This study was an essentially qualitative study conducted in one region of Ghana. Its’ major usefulness is in providing an in-depth understanding of the “how” and “why” of frontline provider decision making in a particular context. It cannot be assumed to be generalizable beyond the study context. However given some contextual similarities across Ghana as well as parts of sub-Saharan Africa and other low and middle income countries – it is possible that similar observations will be made if the study is repeated elsewhere.

## Supporting Information

Box S1 **Frontline provider of maternal and newborn services Evidence Based Tools and Guidelines locally available in Ghana (have been adapted by Ministry of Health (MOH)/Ghana Health Service (GHS) from internationally available guidelines and protocols for specific local use in Ghana).**
(DOC)

## References

[1] WHO (2010) Millennium Development Goals (MGDs) Report, 2010.

[2] Ghana Statistical Service (GSS), Ghana Health Service (GHS), and Macro International (2009) Ghana Maternal Health Survey 2007. Calverton, Maryland, USA: GSS, GHS, and Macro International.

[3] WHO (2012) Trends in Maternal Mortality: 1990 to 2010. WHO, UNICEF, UNFPA and The World Bank Estimates. Available: http://whqlibdoc.who.int/publications/2012/9789241503631_eng.pdf. Accessed 2012 December 17.

[4] UNICEF (2012) Levels and Trends in Child Mortality Report 2012. Estimates developed by the UN Interagency group for child mortality estimates. UNICEF/WHO/World Bank/United Nations. Available: http://childmortality.org/files_v9/download/Levels%20and%20Trends%20in%20Child%20Mortality%20Report%202012.pdf. Accessed 2012 December 17.

[5] World Health Organization and UNICEF (2012) Countdown 2015 Maternal, Newborn and Child survival. Building a future for women and children. The 2012 report. Available: http://www.countdown2015mnch.org/documents/2012Report/2012-complete-no-profiles.pdf. Accessed 2012 December 17.

[6] Kinney MV, Kerber KJ, Black RE, Cohen B, Nkrumah F, et al (2010) Sub-Saharan Africa's Mothers, Newborns, and Children: Where and Why Do They Die?. PLoS Med 7(6): e1000294 doi:10.1371/journal.pmed.1000294. 20574524 10.1371/journal.pmed.1000294 PMC2888581

[7] Bhutta ZA, Chopra M, Axelson H, Berman P, Boerma T, et al.. (2010) Countdown to 2015 Decade Report (2000–10): Taking Stock of Maternal, Newborn, and Child Survival. Lancet 2010. 375: p. 2032–44. 10.1016/S0140-6736(10)60678-2 20569843

[8] Hogan MC, Foreman KJ, Naghavi M, Ahn SY, Wang M, et al.. (2010) Maternal mortality for 181 countries, 1980 – 2008: A Systematic Analysis of Progress Towards Millennium Development Goal 5. Lancet 2010; 375: 1609 – 23. 10.1016/S0140-6736(10)60518-1 20382417

[9] Ghana Statistical Service (GSS), Ghana Health Service (GHS), and ICF Macro (2009) Ghana Demographic and Health Survey 2008. Accra Ghana: GSS, GHS, and ICF Macro.

[10] Penfold S., et al.. (2007), Evaluation of the Delivery Fee Exemption Policy In Ghana: Population Estimates of Changes in Delivery Service Utilization in Two Regions. Ghana Med J, 2007. 41(3): p. 100–9. PMC2279083 18470327

[11] Bosu WK, Bell JS, Armar-Klemesu M, Ansong-Tornui J (2007) Effect of delivery care user fee exemptions policy on institutional maternal deaths in the central and Volta regions of Ghana. Ghana Medical Journal. Vol. 41, No.3 118 – 124. 10.4314/gmj.v41i3.55278 PMC2279091 18470329

[12] Ansong-Tornui J, Armar-Klemesu M, Arhinful D, Penfold S, Hussein J (2007) Hospital Based Maternity Care in Ghana – Findings of a confidential enquiry into maternal deaths. Ghana Medical Journal. Vol. 41, No.3. 125 – 132. 10.4314/gmj.v41i3.55280 PMC2279086 18470330

[13] Witter S (2009) Providing free maternal health care: ten lessons from an evaluation of the national delivery exemption policy in Ghana. Glob Health Action 2009: 2. 10.3402/gha.v2i0.1881 PMC2779941 20027275

[14] Ghana Statistical Service (GSS), Ghana Health Service (GHS), and Macro International. 2009. Ghana Maternal Health Survey 2007. Calverton, Maryland, USA: GSS, GHS, and Macro International.

[15] Ghana Statistical Service (GSS), Ghana Health Service (GHS), and ICF Macro (2009) Ghana Demographic and Health Survey 2008. Accra Ghana: GSS, GHS, and ICF Macro.

[16] UNICEF, UNICEF. Countdown to 2015: tracking progress in maternal, newborn and child survival: 2008 report. 2008, UNICEF: New York.

[17] Deganus S, Tornui J (2006), Impact of free delivery policy on utilization and quality of care at B level (basic obstetric care) facilities in Ghana. 2006, IMMPACT: Aberdeen and Accra.

[18] Kim YM, Kols A, Martin A, Silva D, Rinehart W, et al.. (2005) Promoting Informed Choice: Evaluating a Decision-Making Tool for Family Planning Clients and Providers in Mexico. International Family Planning Perspectives Volume 31, Number 4, December 2005. 10.1363/3116205 16439343

[19] Health Research Unit Ghana Health Service, Frontiers Program Population Council, Prime II IntraHealth International (2005) Improving the Ghanaian Safe Motherhood Program. Evaluating the effectiveness of alternative training models and other performance improvement factors on the quality of maternal care and client outcomes. Available: http://www.popcouncil.org/pdfs/frontiers/FR_FinalReports/Ghana_SM.pdf. Assessed 2013 January 16.

[20] Baker U, Tomson G, Some M, Koyate B, Williams J, et al (2012) “How to know what you need to do”: a cross country comparison of maternal health guidelines in Burkina Faso, Ghana and Tanzania. Implementation Science 7: 31. http://www.implementationscience.com/content/7/1/31. Assessed 2013 January 16. 10.1186/1748-5908-7-31 PMC3372446 22500744

[21] Dopson S, FitzGerald L, Ferlie E, Gabbay J, Locock L (2002) No magic targets! Changing Clinical Practice to become more evidence based. Health Care Manage Rev, 27 (3), 35 – 47. 10.1097/00004010-200207000-00005 12146782

[22] Gabbay J, le May A (2004) Evidence based guidelines or collectively constructed “mindlines”? Ethnographic study of knowledge management in primary care. BMJ 2004 329: 1013 doi:10.1136/bmj.329.7473.1013. 10.1136/bmj.329.7473.1013 PMC524553 15514347

[23] Andre M, Borgquist L, Foldevi M, Molstad S (2002) Asking for ‘rules of thumb’: a way to discover tacit knowledge in general practice. Family Practice. Vol 19 No 6. 617 – 622. 10.1093/fampra/19.6.617 12429664

[24] Ghana Statistical Service (GSS), Ghana Health Service (GHS), and ICF Macro (2009) Ghana Demographic and Health Survey 2008. Accra Ghana: GSS, GHS, and ICF Macro.

[25] Ghana Statistical Service (GSS), Ghana Living Standards Survey, Report of the Fifth Round (GLSS 5), GSS 2008.

[26] Ghana Statistical Services (GSS), Ghana Health Service (GHS), and Marcro International, 2009. Ghana Maternal Health Survey 2007, Calverton, Maryland, USA: GSS, GHS and Macro International.

[27] Ghana Statistical Services (GSS), Ghana Health Service (GHS), and ICF Macro, 2009. Ghana Demographic and Health Survey 2008. Accra, Ghana: GSS, GHS, and ICF Macro.

[28] Gabbay J, le May A (2004) Evidence based guidelines or collectively constructed “mindlines”? Ethnographic study of knowledge management in primary care. BMJ 2004 329: 1013 doi:10.1136/bmj.329.7473.1013. 10.1136/bmj.329.7473.1013 PMC524553 15514347

[29] Miles A, Loughlin M, Polychronis A (2007) Editorial, Introduction and Commentary. Medicine and Evidence: knowledge and action in clinical practice. Journal of Evaluation in Clinical Practice. 13. 481–503. ISSN 1356-1294. 10.1111/j.1365-2753.2007.00923.x 17683283

[30] Dopson S, Fitz Gerald L, Ferlie E, Gabbay J, Locock L (2002) No magic targets! Changing Clinical Practice to become more evidence based. Health Care Management Review. 27 (3) 35–47. 10.1097/00004010-200207000-00005 12146782

[31] Turner TJ (2009) Developing evidence-based clinical practice guidelines in hospitals in Australia, Indonesia, Malaysia, the Philippines and Thailand: values, requirements and barriers. BMC Health Serv Res. 2009 Dec 15 9: 235. 10.1186/1472-6963-9-235 PMC2800111 20003536

[32] Theodorou M, Tsiantou V, Pavlakis A, Maniadakis N, Fragoulakis V, et al (2009) Factors influencing prescribing behaviour of physicians in Greece and Cyprus: results from a questionnaire based survey. BMC Health Serv Res. 2009 Aug 20 9: 150. 10.1186/1472-6963-9-150 PMC2737540 19695079

[33] Wagai J, Senga J, Fegan G, English M (2009) Examining agreement between clinicians when assessing sick children. PLoS One. 2009; 4(2): e4626. Epub 2009 Feb 27. 10.1371/journal.pone.0004626 PMC2644760 19247448

[34] Research and Development Division Ghana Health Service Ethical Clearance - ID NO: GHS-ERC: 02/09/11. Protocol titled: “An Investigation of Availability and use of Clinical Decision Making Support Tools for MDG 4&5 and Provider Decisions Making Processes”.

[35] Maine D (1994) Too far to walk: Maternal mortality in context. Social Science and Medicine 38, 1091 – 1110. 10.1016/0277-9536(94)90226-7 8042057

[36] Zurovac D, Sudoi RK, Akwahle WS, Ndiritu M, Hamer DH, et al (2011) The effect of mobile phone text-message reminders on Kenyan health workers adherence to malaria treatment guidelines: a cluster randomized trial. Available: www.thelancet.com. August 4 2011. 6736 (11) 60783–6. Assessed 2013 January 16. 10.1016/S0140-6736(11)60783-6 PMC3163847 21820166

[37] Noordam AC, Kuepper BM, Stekelenburg J, Milen A (2011) Improvement of maternal health services through the use of mobile phones. Tropical Medicine and International Health. Volume 16 No. 5 622 – 626. 10.1111/j.1365-3156.2011.02747.x 21342374

