# Supplementary material for: Care Decision Making of Frontline Providers of Maternal and Newborn Health Services in the Greater Accra Region of Ghana
Source: PLoS One. 2013 Feb 13;8(2):e55610. doi: 10.1371/journal.pone.0055610 (PMC3572062; doi:10.1371/journal.pone.0055610)
Supplement: Box S1 — Frontline provider of maternal and newborn services Evidence Based Tools and Guidelines locally available in Ghana (have been adapted by Ministry of Health (MOH)/Ghana Health Service (GHS) from internationally available guidelines and protocols for specific local use in Ghana). (DOC) [file pone.0055610.s001.doc]

Box S1 – Frontline provider of maternal and newborn services Evidence Based Tools and Guidelines locally available in Ghana (have been adapted by Ministry of Health (MOH)/Ghana Health Service (GHS) from internationally available guidelines and protocols for specific local use in Ghana)

1. *Ghana Health Service (2008) National safe motherhood (SM) service protocol*. Provides guidance on the provision of effective antenatal, delivery and postnatal care.
2. Ghana Health Service (2007) The *National family planning protocols manual*. Documents available family planning methods*.* Various user friendly job aids, such as flipcharts, counseling cards and leaflets have been adapted from this manual.
3. Reproductive Health Service Policy and Standards (2003) is a protocol on abortion care. The *Ghana standards and protocols on comprehensive abortion care*, is adapted from this manual
4. Ministry of Health – Ghana National Drugs Program (2011) Standard Treatment Guidelines (STG). These have been in use in Ghana for over a decade and are revised periodically. They are generic for clinical care as a whole rather than specific for maternal and newborn care.
5. Local adaptation of the WHO Integrated Management of Neonatal and Childhood Illness (IMNCI) manual and chart booklets by Ghana Health Service. The IMNCI manuals provide guidance on Integrated management of neonatal and childhood illnesses.
6. Ghana Health Service (2006) Prevention and Management of Unsafe Abortion: Comprehensive Abortion Care Services, Standards and Protocol, June 2006. This document is used as a guide to facilitate the provision of Comprehensive Abortion Care services to help reduce unwanted pregnancy and abortion related morbidity and mortality in Ghana.
7. Improving access to quality care in Family Planning (FP), Medical Eligibility Criteria for contraceptive use (MEC Wheel) (2008). It contains information on medical eligibility for starting use of contraceptive methods. It directs family planning providers on which contraceptive method is the safest and most effective for a woman requesting family planning services.

References

1. Ghana Health Service (2008) National Safe Motherhood Service Protocol. Printed in Ghana by Yamens Press Limited, Accra, Ghana, West Africa. P.O. Box AF 274, Adenta-Accra. Tel: +233 302 223222/235036. Email: [yamenspressltd@yahoo.com](mailto:yamenspressltd@yahoo.com)
2. Ghana Health Service (2007) National Family Planning Protocols. Printed in Ghana by Yamens Press Limited, Accra, Ghana, West Africa. P.O. Box AF 274, Adenta-Accra. Tel: +233 302 223222/235036. Email: [yamenspressltd@yahoo.com](mailto:yamenspressltd@yahoo.com)
3. Ministry of Health /Ghana Health Service (2003) Reproductive Health Service Policy and Standards
4. Ministry of Health (GNDP) Ghana (2010) Standard Treatment Guidelines 6^th^ Edition. Ghana National Drugs Program (GNDP), Ministry of Health P.O. Box MB 582 Accra, Ghana. Available: http://ghndp.org/images/downloads/stg2010.pdf
5. World Health Organization (2006) Integrated Management of Neonatal and Childhood Illness chart booklet. WHO. 20 Avenue Appia 1211 Geneva 27, Switzerland
6. Ghana Health Service (2006) Prevention and Management of Unsafe Abortion: Comprehensive Abortion
7. Improving access to quality care in FP, Medical Eligibility Criteria for contraceptive use (MEC Wheel) <http://whqlibdoc.who.int/hq/1996/WHO_FRH_FPP_96.9_eng.pdf>
